# Reduced Proliferation in the Adult Mouse Subventricular Zone Increases Survival of Olfactory Bulb Interneurons

**DOI:** 10.1371/journal.pone.0031549

**Published:** 2012-02-21

**Authors:** Yi Sui, Malcolm K. Horne, Davor Stanić

**Affiliations:** 1 Neurodegeneration Division, Florey Neuroscience Institutes, University of Melbourne, Parkville, Victoria, Australia; 2 Centre for Neuroscience, University of Melbourne, Parkville, Victoria, Australia; 3 Neurology Department, St Vincent's Hospital, Fitzroy, Australia; Université Pierre et Marie Curie-Paris6, INSERM, CNRS, France

## Abstract

Neurogenesis in the adult brain is largely restricted to the subependymal zone (SVZ) of the lateral ventricle, olfactory bulb (OB) and the dentate subgranular zone, and survival of adult-born cells in the OB is influenced by factors including sensory experience. We examined, in mice, whether survival of adult-born cells is also regulated by the rate of precursor proliferation in the SVZ. Precursor proliferation was decreased by depleting the SVZ of dopamine after lesioning dopamine neurons in the substantia nigra compacta with 6-hydroxydopamine. Subsequently, we examined the effect of reduced SVZ proliferation on the generation, migration and survival of neuroblasts and mature adult-born cells in the SVZ, rostral migratory stream (RMS) and OB. Proliferating cells in the SVZ, measured by 5-bromo-2-deoxyuridine (BrdU) injected 2 hours prior to death or by immunoreactivity against Ki67, were reduced by 47% or 36%, respectively, 7 days after dopamine depletion, and by 29% or 31% 42 days after dopamine depletion, compared to sham-treated animals. Neuroblast generation in the SVZ and their migration along the RMS were not affected, neither 7 nor 42 days after the 6-hydroxydopamine injection, since the number of doublecortin-immunoreactive neuroblasts in the SVZ and RMS, as well as the number of neuronal nuclei-immunoreactive cells in the OB, were stable compared to control. However, survival analysis 15 days after 6-hydroxydopamine and 6 days after BrdU injections showed that the number of BrdU+ cells in the SVZ was 70% higher. Also, 42 days after 6-hydroxydopamine and 30 days after BrdU injections, we found an 82% increase in co-labeled BrdU+/γ-aminobutyric acid-immunoreactive cell bodies in the granular cell layer, while double-labeled BrdU+/tyrosine hydroxylase-immunoreactive cell bodies in the glomerular layer increased by 148%. We conclude that the number of OB interneurons following reduced SVZ proliferation is maintained through an increased survival of adult-born precursor cells, neuroblasts and interneurons.

## Introduction

The mammalian nervous system arises from coordinated proliferation, differentiation and migration of precursor cells during embryonic and early postnatal development [1]. Although most of these processes are completed by the perinatal period, neurogenesis continues throughout adulthood in the subventricular or subependymal zone (SVZ) of the lateral ventricle and olfactory bulb (OB) [2], [3], as well as the subgranular zone of the hippocampal dentate gyrus [4], [5].

**Figure 1:**
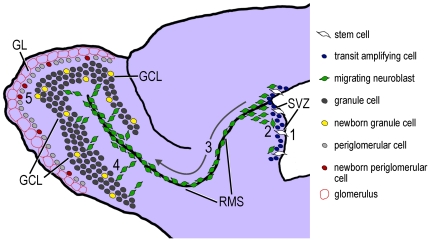
Neurogenesis in the adult rodent SVZ and OB. Schematic sagittal view of the adult mouse brain. 1) Adult-born olfactory precursors (stem cells/transit amplifying cells) proliferate primarily within the SVZ where they 2) differentiate into immature neurons (neuroblasts). 3) Neuroblasts then migrate tangentially along the RMS toward the main OB, which requires 2–6 days. Arrow indicates the direction of neuroblast migration through the RMS. 4) On days 5–7 after birth, adult-born neuroblasts migrate radially towards the granular, periglomerular and external plexiform cell layers of the OB. 5) 15–30 days after birth, adult-born cells in the OB mature to form local interneurons that display extensive dendritic arborizations (see [9], [10], [32]). GCL, granular cell layer; GL, glomerular layer; RMS, rostral migratory stream; SVZ, subependymal zone.

Adult olfactory precursors divide primarily within the SVZ, where they differentiate into immature neurons. Neuroblasts then migrate tangentially along the rostral migratory stream (RMS) toward the main OB. When neuroblasts reach the OB, they migrate radially into the granular (GCL), periglomerular (GL) and external plexiform cell layers of the OB, and differentiate into local interneurons [3], [6], [7], [8] (Fig. 1).

Over recent decades, a wide range of molecular cues have been identified to regulate neurogenesis during development, many of which continue to influence neurogenesis in the adult [9], [10], [11], [12]. The neurotransmitter dopamine (DA), for example, modulates the cell cycle of lateral ganglionic eminence progenitors during development through DA D1- and D2-like receptors [13]. In the adult brain, dopaminergic (DAergic) fibers innervate the SVZ and provide afferents to transit amplifying cells that express D1- and D2-like receptors [14], [15]. Proliferation of these and stem cells in the adult SVZ is under the influence of D2-like receptors [14], [16], [17], and depletion of DA innervation to the SVZ reduces the number of proliferating cells in the SVZ [14], [15], [18], [19]. More recent studies have identified that DA-induced proliferation of transit amplifying cells in the adult SVZ is mediated through epidermal growth factor (EGF) and EGF receptor stimulation [20], and ciliary neurotrophic factor [21].

Among the molecules governing migration of neuroblasts through the RMS are members of the ephrin-B family [22], glial cell line-derived neurotrophic factor [23], [24], the polysialylated form of the neural-cell adhesion molecule [25], [26] and doublecortin (DCX). DCX, a neuron specific microtubule associated protein, is expressed on the cell body and leading processes of most migrating postmitotic neuroblasts, and directs migration by regulating the organization and stability of microtubules that make up the cytoskeleton of neuroblasts [27], [28], [29]. DCX is expressed by dividing neuroblast cells in the SVZ, and newly generated neuroblasts continue to express DCX as they migrate through the RMS and enter the OB [30]. Downregulation of DCX begins 10–14 days after the birth of a neuroblast, and coincides with the commencement of neuronal nuclei (NeuN) expression, as the cells mature to become OB interneurons [30].

In the young adult, approximately 50% of adult-born cells that migrate into the OB differentiate to form interneurons that integrate into OB circuitry, while the other half undergo programmed cell death as progenitors, neuroblasts or young neuronal cells in the SVZ, RMS or OB [31], [32], [33]. At the molecular level, noradrenergic [34], [35] and cholinergic transmission [36], [37], [38], as well as cAMP response element binding protein (CREB) signaling, are thought to play important roles in regulating survival and death of adult-born cells residing in the OB. Disruption of CREB signaling reduces the survival of newborn neurons in the OB [39] and inhibits expression of the neurogenic transcription factor Pax 6 [40]. Pax 6 regulates the survival of mature OB DA neurons by controlling their expression of crystalline αA, which prevents apoptosis by inhibition of procaspase-3 activation [41]. At a more integrated level, survival of adult-born OB cells is associated with sensory experience [42]. Enriched odor exposure [43] and learning of olfactory discrimination [44] and associative [45] tasks are among the factors that promote survival, and this depends on the age of the cell and its location in the OB [46]. Sensory deprivation, on the other hand, decreases survival of new granule cells [47], [48].

While there is increasing understanding of specific factors that control proliferation, migration and survival of adult-born cells in the OB, it still remains unclear whether the rate of neuronal precursor generation in the SVZ influences the longevity of differentiated precursors integrated in the OB. The proliferative rate of precursors in the SVZ and survival of cells in the OB appears linked because the volume of the OB does not substantially change throughout life [32], [49], [50], [51]. To establish if this is the case, we experimentally reduced the number of proliferating precursor cells in the mouse SVZ, and analyzed neuroblast formation in the SVZ, neuroblast migration through the RMS, as well as the phenotype and survival of OB interneurons.

## Results

The effect of reduced cell proliferation in the SVZ of the adult mouse on the generation of neuroblasts in the SVZ, their migration through the RMS, and their survival in the OB was examined. The rate of precursor cell proliferation in the SVZ was lowered by reducing DA innervation to the striatum and SVZ [14]. This was achieved by injecting 6-hydroxydopamine-hydrobromide (6-OHDA) into the substantia nigra pars compacta (SNc) to destroy local DA-producing neurons and their nigrostriatal projections. We then performed stereological quantification to examine whether reduced SVZ proliferation altered: 1) the number of neuroblasts generated in the SVZ and RMS; 2) the number of neuroblasts migrating through the RMS; 3) the number of interneurons present in the OB; and 4) the survival of newborn cells in the OB. Results from individual experiments are presented first, followed by a summary of results in Table 1.

**Table 1:** Summary of results, demonstrating the change in the number of cell bodies in the SVZ, RMS, GCL and GL of 6-OHDA injected animals when compared to control.

Subventricular Zone								
Protocol	BrdU	Ki67	DCX					
1	↓/↓ (3A)	↓/↓ (3C)	= / = (4A)					
2	↑ (4C,E)							
3								

Rostral Migratory Stream								
Protocol	BrdU	Ki67	DCX					
1	= / = (3B)	= / = (3D)	= / = (4B)					
2	= (4D,E)							
3								

Granular Cell Layer								
Protocol	BrdU	BrdU/GABA	GABA	CalR	NeuN			
1			/ = (6A)	/ = (6D)	= / = (3K)			
2								
3	↑ (5A)	↑ (5D)						

Glomerular Layer								
Protocol	BrdU	BrdU/TH	GABA	TH	GABA/TH	CalR	CalB	NeuN
1			= / = (6G)	↑/↑ (6H)	↑/↑ (6I)	= /↓ (6M)	= / = (6N)	= / = (3L)
2								
3	↑ (5H)	↑ (5L)						

**Figure 2:**
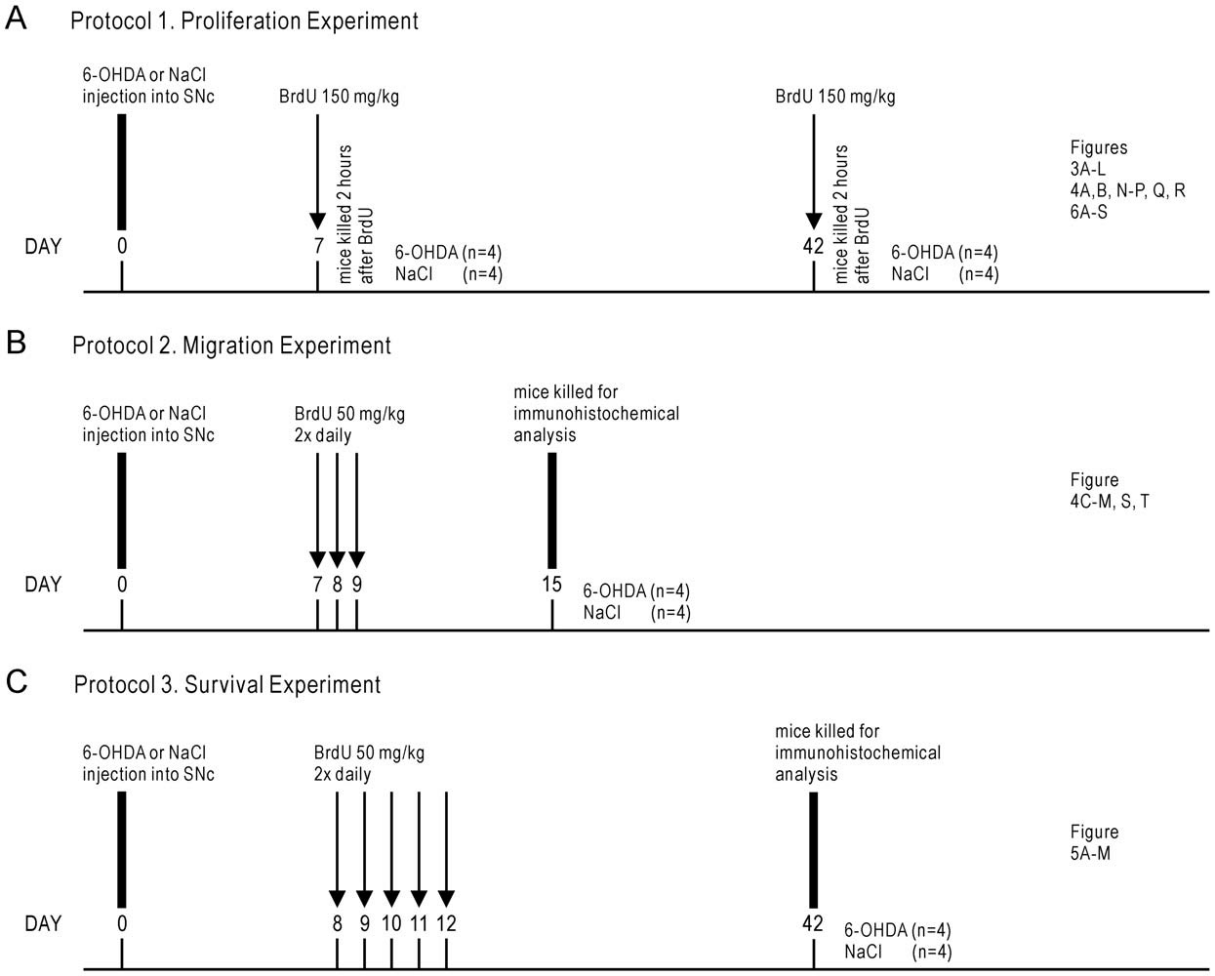
Experimental design. At the beginning of each experiment (Day 0), mice received injections of either 6-OHDA or 0.9% NaCl (control) into the SNc. (A) *Protocol 1*. To identify proliferating cells in the SVZ and RMS, a single dose of BrdU (150 mg/kg i.p.) was administered 2 hours prior to death, 7 or 42 days after the 6-OHDA or NaCl injections (n = 4 for each experimental group). From these animals, the number of BrdU+ (Fig. 3A, B, E–G), Ki67-ir (Fig. 3C, D, H–J) and DCX-ir (Fig. 4A, B, N–P) cell bodies in the SVZ and RMS were estimated, as were the number of GFAP-ir glial cells in the SVZ (Fig. 4Q, R), and the number of NeuN-ir (Fig. 3K, L), GABA-ir (Fig. 6A–C, G–L), calretinin-ir (Fig. 6D–F, M, O, P), TH-ir (Fig. 6H–J) and calbindin-ir (Fig. 6N, Q–R) cell bodies in the OB. (B) *Protocol 2*. To identify migrating neuroblasts in the RMS and the extent of their migration along the RMS, BrdU (50 mg/kg, i.p.) was administered twice daily for 3 consecutive days beginning 7 days after 6-OHDA (n = 4) or NaCl (n = 4) injections into the SNc, and mice were killed 6 days later (i.e. 15 days after 6-OHDA administration). From these animals, the number of BrdU+ and DCX-ir cell bodies in the SVZ and RMS were quantified (Fig. 4C–M, S, T). (C) *Protocol 3*. To label mature adult-born cells that migrate to, integrate and survive in the GCL and GL, BrdU (50 mg/kg, i.p.) was administered twice daily for 5 consecutive days, beginning 8 days after 6-OHDA (n = 4) or NaCl (n = 4) was injected into the SNc, and mice killed 30 days later (i.e. 42 days after 6-OHDA administration). From these animals, the number of BrdU+ cell bodies in the GCL (Fig. 5A–G) and GL (Fig. 5H–M) were estimated, as were the number of BrdU/GABA co-expressing cells in the GCL (Fig. 5D–G), and the number of BrdU/TH co-expressing cells in the GL (Fig. 5K–M).

*Protocol 1*. A single dose of BrdU (150 mg/kg i.p.) was administered 2 hours prior to death, 7 or 42 days after the 6-OHDA or NaCl injections (n = 4 for each experimental group) (see Fig. 2A). First symbol represents 7 day group; second symbol corresponds to 42 day group. *Protocol 2*. BrdU (50 mg/kg, i.p.) was administered twice daily for 3 consecutive days beginning 7 days after 6-OHDA (n = 4) or NaCl (n = 4) injections into the SNc, and mice killed 6 days later (i.e. 15 days after 6-OHDA administration) (see Fig. 2B). *Protocol 3*. BrdU (50 mg/kg, i.p.) was administered twice daily for 5 consecutive days, beginning 8 days after 6-OHDA (n = 4) or NaCl (n = 4) was injected into the SNc, and mice killed 30 days later (i.e. 42 days after 6-OHDA administration) (see Fig. 2C). ↓ corresponds to reduced number of cell bodies in comparison to control; ↑ corresponds to increased number of cell bodies;  = corresponds to no statistical change in number of cell bodies. Numbers and letters in brackets refer to corresponding figure.

### 6-OHDA injections into the SNc decreases striatal/SVZ DA innervation

Reduced striatal/SVZ DA innervation following 6-OHDA administration to the SNc was confirmed by immunohistochemistry for tyrosine hydroxylase (TH), performed on striatal sections of lesioned animals. 7 or 42 days after 6-OHDA administration there were only occasional TH-immunoreactive (ir) fibers present in the SVZ of lesioned animals (Fig. S1).

### Reduced DA innervation lowers the number of proliferating cells in the SVZ

**Figure 3:**
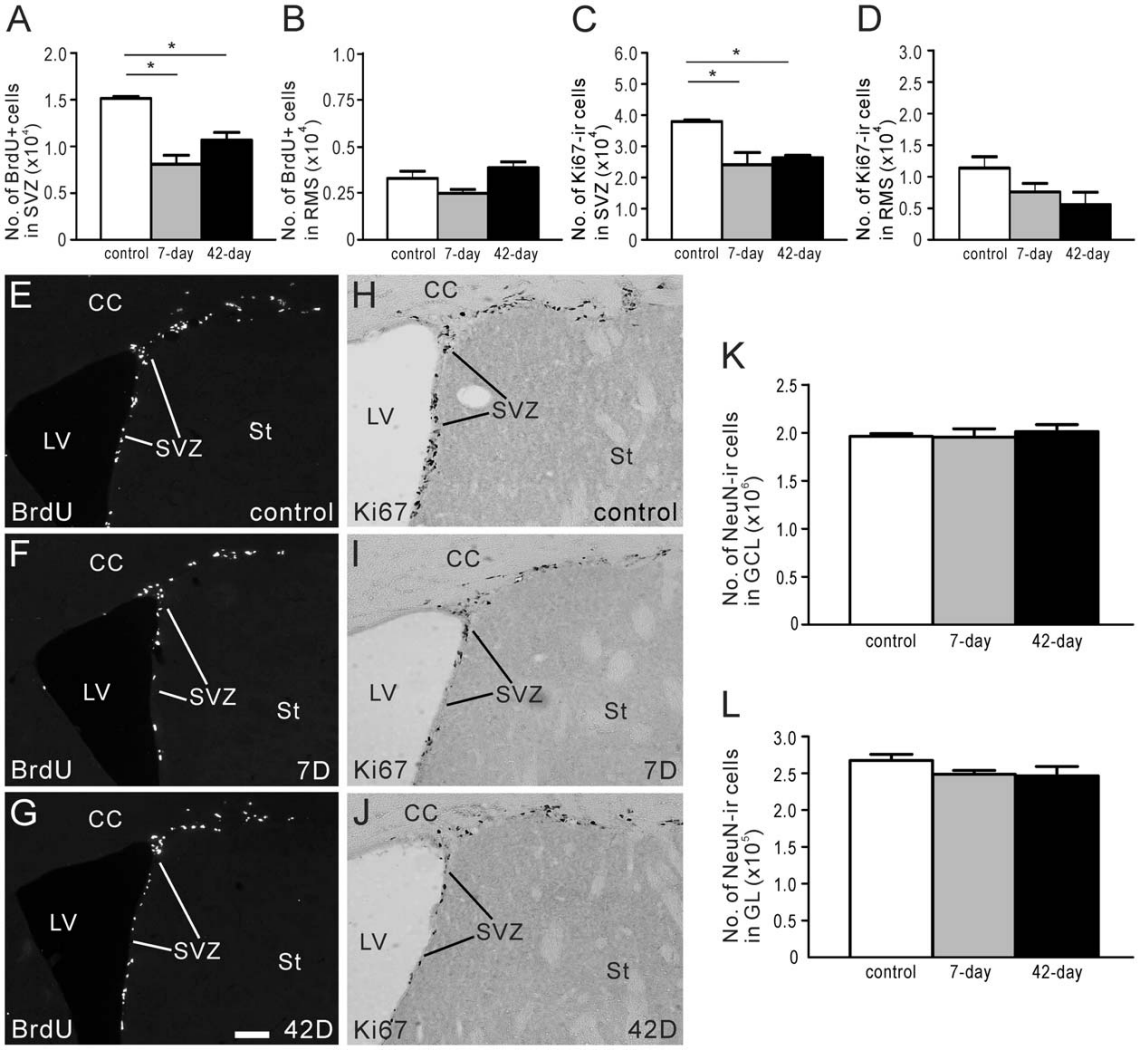
Proliferation in the SVZ and RMS following 6-OHDA injections into the SNc. BrdU was administered 2 hours before animals were killed (see *Protocol 1*, Fig. 2A), and BrdU+ cells in the SVZ and RMS were quantified. (A–D) Estimated number of BrdU+ (A) and Ki67-ir (C) cell bodies in the SVZ. Estimated number of BrdU+ (B) and Ki67-ir (D) cell bodies in the RMS. (E–G) Photomicrographs of BrdU-LI in the SVZ of control mice (E), and mice 7 (F) or 42 days (G) after 6-OHDA was administered to deplete striatal DA. (H–J) Photomicrographs of Ki67-LI in the SVZ of control mice (H), and mice 7 (I) or 42 days (J) after striatal DA denervation. (K, L) Estimated number of NeuN-ir cell bodies in the GCL (K) and GL (L) of control mice, and mice 7 or 42 days after 6-OHDA administration. In plots (A–D) and (K), (L), white bars = control animals (n = 4), grey bars = mice 7 days after 6-OHDA injection (n = 4), and black bars = 42 days after 6-OHDA administration (n = 4). Control animals received injections of 0.9% NaCl into the SNc. CC, corpus callosum; LV, lateral ventricle; St, striatum; SVZ, subventricular zone; 7D, 7 days post 6-OHDA injection; 42D, 42 days post 6-OHDA injection. Scale bar in G = 50 µm, applies E–J. * corresponds to *P*<0.05.

To examine the effect of reduced striatal/SVZ DA innervation on cell proliferation in the SVZ and RMS, 5-bromo-2-deoxyuridine (BrdU) (150 mg/kg i.p.) was injected in mice 2 hours prior to death to label cells in S-phase of the cell cycle (see Fig. 2A). 7 days after administration of 6-OHDA, the number of BrdU-positive (BrdU+) cell bodies in the SVZ was 47% lower than the number in control mice injected with 0.9% sodium chloride (NaCl) (Fig. 3A, E, F), while in the RMS, the 25% decrease in BrdU+ cells was not statistically different from control (Fig. 3B). 42 days after lesioning, the number of BrdU+ cell bodies in the SVZ were down 29% (Fig. 3A, E, G), while in the RMS, the 17% increase in BrdU+ cells was not statistically different from control (Fig. 3B). There was a 37% increase in BrdU+ proliferating cells between 7 and 42 days after 6-OHDA injections were performed (Fig. 3A, B), suggesting a partial recovery in cell proliferation over time following disruption of striatal/SVZ DA innervation.

Immunoreactivity against Ki67 labels cells in all phases of mitosis, except G1, and was used to identify dividing cells in the SVZ and RMS. In the SVZ, Ki67-ir cell bodies were down 36% and 31% 7 and 42 days, respectively, after 6-OHDA was injected into the SNc (Fig. 3C, H–J), while in the RMS the 33% and 51% reduction in Ki67-ir cells, respectively, was not statistically different from control (Fig. 3D).

Although proliferating cells in the SVZ and RMS were reduced by a similar proportion, there were far more dividing cells in the SVZ, so in absolute numbers, the greatest reduction in BrdU+ and Ki67-ir proliferating cells occurred in the SVZ (Fig. 3A cf. 3B, 3C cf. 3D).

### Reduced precursor proliferation in the SVZ has no effect on the number of mature neurons in the OB

As cells born in the SVZ migrate along the RMS toward the OB, where they differentiate into local interneurons [3], [6], [7], [8], we examined the effect that reduced proliferation in the SVZ had on the number of mature interneurons in the OB. Mature OB interneurons were identified using an antibody against NeuN. Despite the reduction in precursor proliferation in the SVZ, the number of NeuN-ir cell bodies in the GCL and GL of the OB were unchanged 7 or 42 days after injection of 6-OHDA into the SNc (Fig. 3K, L).

### Reduced SVZ cell proliferation has no overall effect on the number of DCX-ir neuroblasts in the SVZ and RMS

**Figure 4:**
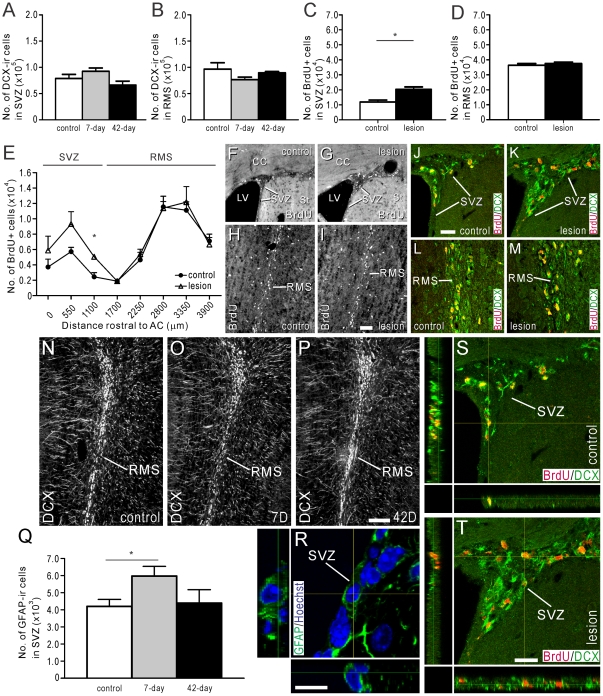
Effect of reduced proliferation in the SVZ on the generation of neuroblasts and their migration through the RMS. (A, B) Estimated number of DCX-ir neuroblasts in the SVZ (A) or RMS (B) of control mice (white bars), and mice 7 (grey bars) or 42 days (black bars) after 6-OHDA injections were performed to lesion the SNc (see *Protocol 1*, Fig. 2A). (C–M, S, T) BrdU (50 mg/kg i.p.) was administered twice daily for 3 consecutive days, beginning 7 days after 6-OHDA or sham injections, and mice were killed 6 days after the last BrdU administration (see *Protocol 2*, Fig. 2B). (C, D) Estimated number of BrdU+ cell bodies in the SVZ (C) or RMS (D) of control mice (white bars), and mice 15 days after 6-OHDA was injected (black bars). (E) Estimated number of BrdU+ cell bodies in the RMS of control or 6-OHDA injected mice, plotted according to distance rostral to the AC. Note the increase in BrdU+ cells in the SVZ of 6-OHDA injected animals. Photomicrographs showing BrdU-LI in the SVZ (F, G) and RMS (H, I) of control and 6-OHDA injected mice. Double-immunofluorescence confocal micrographs of BrdU- (red) and DCX-LI (green) in the SVZ (J, K) and RMS (L, M) of control (J, L) and 6-OHDA injected (K, M) mice. (S, T) Double-immunofluorescence confocal micrographs of BrdU- (red) and DCX-LI (green) in the SVZ of control (S) and 6-OHDA injected (T) mice, shown at higher magnification. Note increased number of double-labeled cells in the SVZ of 6-OHDA injected mice. (N–P) Photomicrographs of DCX-ir neuroblasts in the RMS of control mice (N), and 7 (O) or 42 days (P) after 6-OHDA injection into the SNc (see *Protocol 1*, Fig. 2A). (Q, R) Estimated number of GFAP-ir astrocytes in the SVZ of control mice (white bar), and 7 (grey bar) or 42 days (black bar) after 6-OHDA administration (Q) (see *Protocol 1*, Fig. 2A). (R) Double-immunofluorescence confocal micrograph of GFAP-LI (green) and Hoechst staining (blue) in the SVZ, the later providing a nuclear counter stain. In plots (A), (B) and (Q), white bars = control animals (n = 4), grey bars = mice 7 days after 6-OHDA injection (n = 4), and black bars = 42 days after 6-OHDA administration (n = 4). For control and lesion groups in plots C–E, n = 4. Control animals received injections of 0.9% NaCl into the SNc. Scale bars: I = 50 um, applies F–I; J = 20 um, applies J–M; P = 100 um. applies N–P; R = 10 um; T = 20 um, applies S, T. * corresponds to *P*<0.05.

Because reduced cell proliferation in the SVZ did not alter the total number of mature OB neurons, we next examined whether the number of neuroblasts in the SVZ and RMS were altered. Neuroblasts in these regions were identified by immunoreactivity against DCX [27], [28], [30]. We found that the number of DCX-ir neuroblasts in the SVZ and RMS 7 and 42 days after 6-OHDA was injected into the SNc was similar to control animals (Fig. 4A, B, N–P).

### Impact of reduced SVZ proliferation on migration of neuroblasts through the RMS

The effect of reduced precursor proliferation in the SVZ on the migration of neuroblasts through the RMS was examined by administering BrdU (50 mg/kg i.p.) twice daily for 3 consecutive days, beginning 7 days after 6-OHDA (or sham) injections. The mice were killed 6 days after the last BrdU administration to allow quantification of the distance that neuroblasts had migrated through the RMS [33] (see Fig. 2B).

6 days after the last BrdU administration (i.e. 15 days after 6-OHDA administration), the number of BrdU+ cell bodies in the SVZ increased by 70% in lesioned mice (Fig. 4C), while in the RMS, where most BrdU+ cells were located, the 3% increase in lesioned mice was not statistically different from control (Fig. 4D).

When the number of BrdU+ cell bodies was plotted against distance rostral to the convergence of the anterior commissure (Fig. 4E), it was apparent that the number of BrdU+ cell bodies along the RMS (i.e. 1700–4000 µm rostral to the anterior commissure) was similar in control mice and mice administered with 6-OHDA (Fig. 4E, H, I). Thus, the rate of migration through the RMS, measured by the distance travelled by BrdU+ cells from the SVZ, was similar in mice with reduced proliferation in the SVZ and controls. In contrast, 6 days after the last BrdU administration, there were consistently greater numbers of BrdU+ cell bodies throughout the SVZ of 6-OHDA injected mice (i.e. 0–1100 µm rostral to the anterior commissure) than in control animals, the increase ranging from 39–106% at the 3 points examined in the SVZ (Fig. 4E–G).

### An increased number of BrdU+ cell bodies in the SVZ corresponds with a higher number of DCX-ir neuroblasts

Double-immunofluorescence histochemistry for BrdU and DCX was performed to reveal the identity of the BrdU+ cell bodies in the SVZ. 6 days after the last administration of BrdU to control mice or those that received 6-OHDA injections, most BrdU+ cell bodies in the SVZ co-expressed DCX-like immunoreactivity (LI) (Fig. 4J, K, S, T). Together with the 70% increase in the number of BrdU+ cell bodies observed in the SVZ of 6-OHDA injected mice (Fig. 4C), our data suggest that BrdU+ cells in the SVZ 6 days after BrdU administration were DCX-ir neuroblasts.

### Impact of reduced striatal/SVZ DA innervation on GFAP-ir astrocytes in the SVZ

We also performed immunohistochemistry for glial fibrillary acidic protein (GFAP, Fig. 4R) to determine whether reduced cell proliferation in the SVZ had an effect on the number of astrocytes [52]. There were 42% more GFAP-ir astrocytes in the SVZ 7 days after 6-OHDA administration compared to control mice (Fig. 4Q). 42 days after 6-OHDA was injected into the SNc, the 5% increase of GFAP-ir astrocytes in the SVZ was not significantly different from control (Fig. 4Q).

### The number of newborn cells in the OB increases when precursor proliferation in the SVZ is reduced

**Figure 5:**
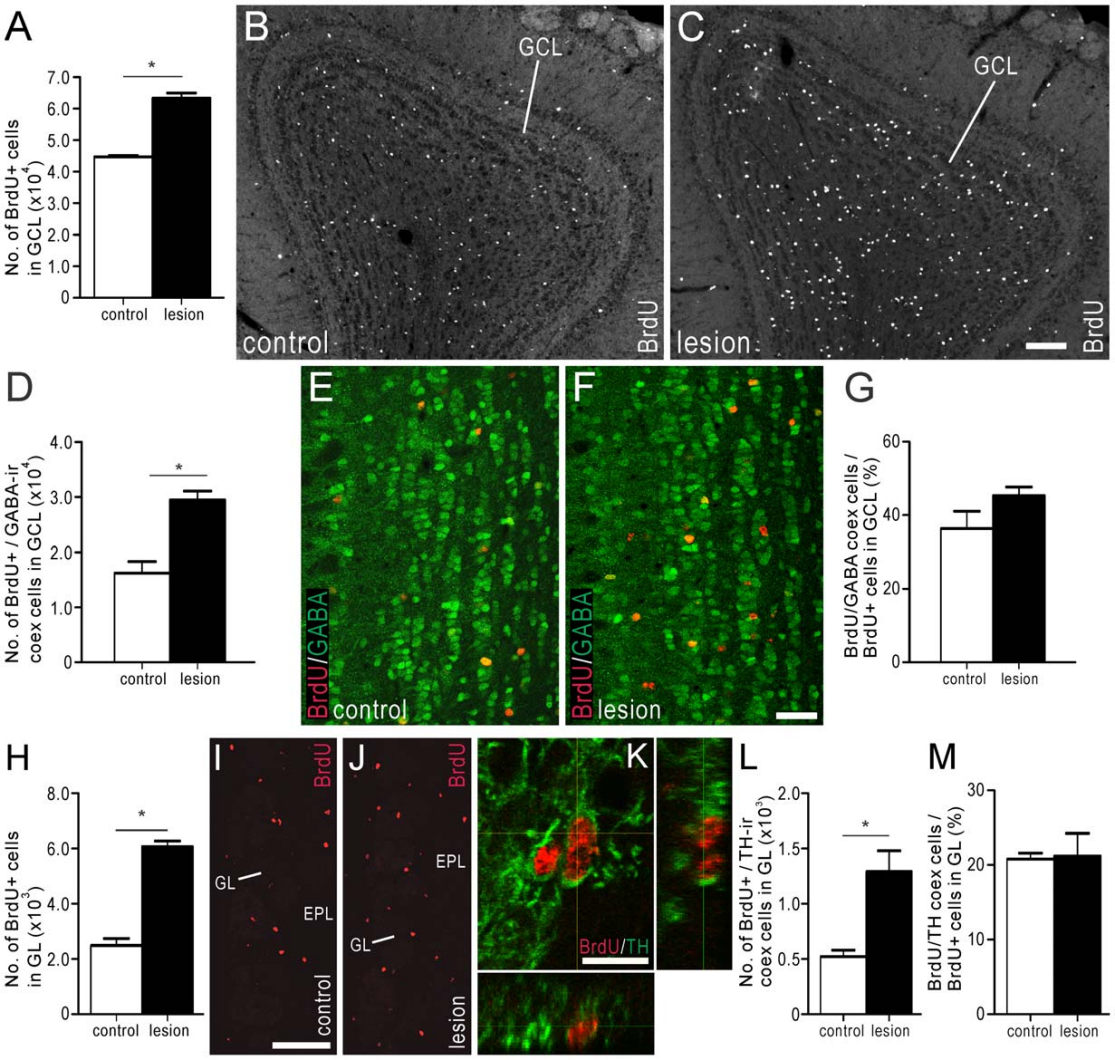
The number of adult-born cells in the OB increases when precursor proliferation in the SVZ is reduced. BrdU (50 mg/kg i.p.) was administered twice daily for 5 consecutive days, beginning 8 days after 6-OHDA (or sham) injection, and mice killed 30 days after the last BrdU administration (i.e., 42 days after 6-OHDA injection; see *Protocol 3*, Fig. 5C). (A–G) Estimated number of BrdU+ cell bodies in the GCL of control (white bar) and 6-OHDA injected mice (black bar) (A). Photomicrograph of BrdU+ cell bodies in the GCL of control (B) and 6-OHDA injected (C) mice. (D) Estimated number of BrdU+ and GABA-ir double-labeled cell bodies in the GCL of control (white bar) and 6-OHDA injected mice (black bar). Photomicrograph of BrdU- (red) and GABA-LI (green) in the GCL of control (E) and 6-OHDA injected (F) mice. (G) Estimated number of BrdU+/GABA-ir co-expressing cell bodies expressed as a proportion of the total number of BrdU+ cell bodies in the GCL of control (white bar) and 6-OHDA injected mice (black bar). (H–M) Estimated number of BrdU+ cell bodies in the GL of control (white bar) and 6-OHDA injected mice (black bar) (H). Photomicrograph of BrdU+ cell bodies in the GL and EPL of control (I) and 6-OHDA injected (J) mice. (K) Double-immunofluorescence confocal micrographs of BrdU- (red) and TH-LI (green) in the GL of mouse with striatal/SVZ DA-depletion. (L) Estimated number of BrdU+ and TH-ir double labeled cell bodies in the GL of control (white bar) and 6-OHDA injected mice (black bar). (M) Estimated number of BrdU+/TH-ir co-expressing cell bodies expressed as a proportion of the total number of BrdU+ cell bodies in the GL of control (white bar) and 6-OHDA injected mice (black bar). In plots (A), (D), (G), (H), (L), (M), white bars = control animals (n = 4), and black bars = 6-OHDA-injected animals (n = 4). Control animals received injections of 0.9% NaCl into the SNc. In (G), (L) and (M) coex = co-expressing. Scale bars: C = 100 um, applies B, C; F = 40 um, applies E, F; I = 100 um, applies I, J; K = 10 um. * corresponds to *P*<0.05.

We next examined the effect of reduced SVZ precursor proliferation on the number of newborn cells that migrate to, integrate and survive in the GCL and GL of the OB. BrdU (50 mg/kg i.p.) was administered twice daily for 5 consecutive days, beginning 8 days after 6-OHDA (or sham) injections. The mice were killed 30 days after the last BrdU administration, a suitable period for assessing the number of newly born cells that have matured and survived in the OB [31], [32], [33] (see Fig. 2C). In the GCL, the number of BrdU+ cell bodies observed in mice administered with 6-OHDA was 42% greater than in control mice (Fig. 5A–C).

Because interneurons in the GCL predominantly express γ-aminobutyric acid (GABA), double-immunofluorescence histochemistry for GABA and BrdU was performed to examine whether reduced SVZ proliferation led to a change in the number of BrdU+ newborn cells that had differentiated into GABA-ir interneurons. 30 days after the last BrdU administration, the number of cells in the GCL that were BrdU+ and contained GABA-LI was 82% greater in 6-OHDA-injected mice than in control mice (Fig. 5D–F). Although the proportion of BrdU+ cell bodies in the GCL that co-expressed GABA-LI increased from 36% in control mice to 45% in mice administered with 6-OHDA (Fig. 5G), this change was not statistically different.

In the GL, the number of BrdU+ cell bodies observed in mice following 6-OHDA administration was 143% greater than in control mice (Fig. 5H–J). Double-immunofluorescence histochemistry for BrdU and TH was then performed (Fig. 5K) to determine whether the increased number of BrdU+ cells in the GL was associated with a rise in DAergic interneurons. We found that the number of BrdU+ cells in the GL that co-labeled TH-LI increased by 148% in mice administered with 6-OHDA when compared with control (Fig. 5L). In the GL of control mice, 21% of BrdU+ cells co-expressed TH-LI, which was similar to the proportion of BrdU+ cells co-expressing TH-LI in mice that received 6-OHDA (Fig. 5M).

### The effect of reduced precursor proliferation in the SVZ on the subclasses of interneurons in the OB

**Figure 6:**
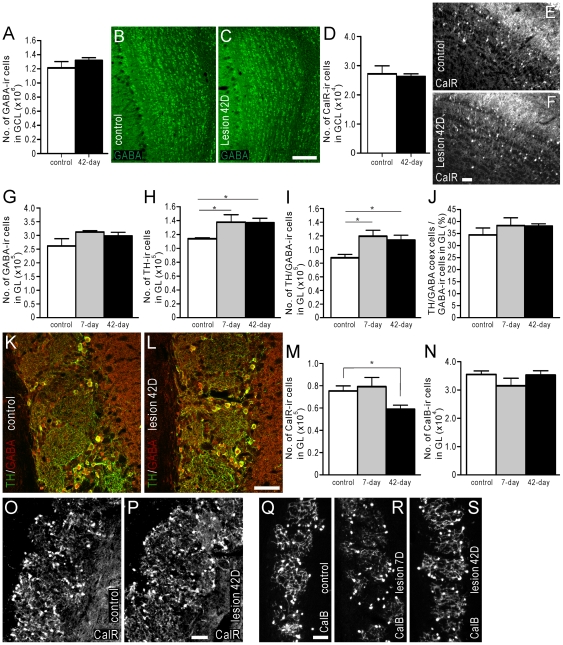
The effect of reduced precursor proliferation in the SVZ on subclasses of interneurons in the GCL and GL. The number of interneurons in the GCL and GL were estimated in animals administered with BrdU 2 hours prior to death (see *Protocol 1*, Fig. 2A). (A–C) Estimated number of GABA-ir cell bodies in the GCL of control mice (white bar), and mice 42 days after 6-OHDA injection (black bar) (A). Photomicrographs of GABA-LI in the GCL of control mouse (B) and 42 days after 6-OHDA injection (C). (D–F) Estimated number of calretinin-ir cell bodies in the GCL of control mice (white bar), and mice 42 days after 6-OHDA injection (black bar) (D). Photomicrographs of calretinin-LI in the GCL of control mouse (E) and 42 days after 6-OHDA injection (F). Estimated number of GABA-ir (G) and TH-ir (H) cell bodies in the GL of control mice, and mice 7 or 42 days following 6-OHDA injection. (I) Estimated number of GABA and TH double-labeled cell bodies in the GL of control mice, and mice 7 or 42 days after 6-OHDA injection. (J) Estimated number of GABA-ir/TH-ir co-expressing cell bodies expressed as a proportion of the total number of GABA-ir cell bodies in the GL of control mice, and mice 7 or 42 days after 6-OHDA injection. (K, L) Double-immunofluorescence photomicrographs of TH- (green) and GABA-LI (red) in the GL of control mice (K), and mice 42 days after 6-OHDA injection (L). Estimated number of calretinin-ir (M) and calbindin-ir (N) cell bodies in the GL of control mice, and mice 7 or 42 days after 6-OHDA injection. (O, P) Photomicrographs of calretinin-LI in the GL of control mice (O), and mice 42 days after 6-OHDA injection (P). (Q–S) Photomicrographs of calbindin-LI in the GL of control mice (Q), and mice 7 (R) or 42 (S) days after 6-OHDA injection. In plots (A) and (D), white bars = control animals (n = 4), and black bars = 6-OHDA-injected animals (n = 4). In plots (G–J), (M), and (N), white bars = control animals (n = 4), grey bars = mice 7 days after 6-OHDA injection (n = 4), and black bars = 42 days after 6-OHDA injection (n = 4). Control animals received injections of 0.9% NaCl into the SNc. In (J) coex = co-expressing. CalR = calretinin; CalB = calbindin. Scale bars: C = 100 um, applies B, C; F = 50 um, applies E, F; L = 50 um, applies K, L; P = 50 um, applies O, P; Q = 50 um, applies Q–S. * corresponds to *P*<0.05.

Several subclasses of granular and periglomerular cells exist in the OB, identified by expression of GABA, TH [53], calbindin and calretinin [54], [55], [56]. We examined whether reduced proliferation in the SVZ affected the number of interneurons in each of these subclasses. In the GCL, the number of GABA-ir (Fig. 6A–C) or calretinin-ir cell bodies (Fig. 6D–F) was similar in control mice, and in mice 42 days after 6-OHDA administration.

In the GL, the number of GABA-ir cell bodies in mice 7 or 42 days after 6-OHDA administration was greater than, but not statistically different from control (Fig. 6G, K, L). In contrast, the 21% and 20% increase in the number of TH-ir cell bodies in the GL 7 and 42 days after 6-OHDA injection, respectively, was statistically different from control mice (Fig. 6H, K, L). Double-labeling experiments revealed that the number of cells co-expressing GABA- and TH-LI increased 36% and 29% in mice 7 and 42 days after 6-OHDA injection, respectively (Fig. 6I, K, L). Despite the increased number of double-labeled GABA/TH cell bodies, the proportion of GABA-ir cells in the GL that co-expressed TH-LI was similar in control mice and mice 7 and 42 days after 6-OHDA administration (Fig. 6J).

In the GL, the number of calretinin-ir cell bodies was similar to control in mice 7 days after 6-OHDA injection. However, in mice 42 days after 6-OHDA administration, 21% fewer calretinin-ir cell bodies were observed (Fig. 6M, O, P). Finally, the number of calbindin-ir cell bodies in the GL of mice 7 and 42 days following 6-OHDA injection was not different from control mice (Fig. 6N, Q–S).

## Discussion

We examined the influence of a reduced number of proliferating precursor cells in the adult SVZ on the generation of neuroblasts, their migration through the RMS and their survival in the OB. Proliferating precursor cells in the SVZ of adult mice were reduced by injecting 6-OHDA to lesion DA cells in the SNc and deplete DAergic afferents that innervate the striatum and SVZ [14], [19]. Our data indicate that reducing precursor proliferation in the SVZ had no effect on the total number of mature neurons in the OB because: 1) the number of NeuN-ir cell bodies in the GCL and GL was similar to control 7 or 42 days after 6-OHDA was injected into the SNc; 2) The number of migrating neuroblasts and DCX-ir neuroblasts in the RMS and SVZ was not changed 7 or 42 days after 6-OHDA administration; and 3) the rate of neuroblast migration through the RMS was similar to control in mice with reduced proliferation in the SVZ. However, the numbers of both BrdU+ neuroblasts in the SVZ and BrdU+ adult-born mature neurons in the OB 15 and 42 days after 6-OHDA injection, respectively, were increased, suggesting that newborn cells have the capacity to survive for a greater period than normal when proliferation in the SVZ is reduced.

Previous studies demonstrated that striatal DA denervation reduces the number of proliferating cells in the SVZ. Treating mice with 1-methyl-4-phenyl-1,2,3,6-tetrahyropyridine (MPTP) depleted striatal DA innervation and reduced the number of SVZ cells expressing the cell cycle marker proliferating cell nuclear antigen (PCNA) by ∼45% and ∼30% 1 and 7 days after administration, respectively [14]. Similarly, 14 [19] and 42 days [14] after administration of 6-OHDA into the rat nigrostriatal pathway, depletion of striatal DA reduced the number of PCNA+ cells in the SVZ by ∼35%. In this study we used the nuclear protein Ki67, which labels cells in all phases of mitosis except G1 [57], to identify proliferating cells. In line with previous reports, our results show a reduction in the number of Ki67-ir cell bodies in the SVZ of 36% and 31%, respectively, 7 and 42 days after 6-OHDA administration. Proliferating cells were also identified by administering BrdU, which incorporates into DNA of dividing cells during the S-phase of the cell cycle [58], to mice with striatal/SVZ DA-depletion 2 hours prior to their death. We found 47% fewer BrdU+ cell bodies in the SVZ 7 days after 6-OHDA administration, and 29% less after 42 days. Thus, our experiments have used a robust model of reduced SVZ proliferation.

Our results point to a regulation of neurogenesis in the olfactory system, so that a steady neuronal population in the OB is maintained. Despite a reduction in proliferating cells in the SVZ caused by 6-OHDA, the number of neuroblasts in the SVZ and RMS was unaltered, and tangential migration of neuroblasts in the RMS was maintained [6]. In addition, 6 days after the last BrdU pulse, the number of BrdU+ cell bodies in the SVZ was increased despite a reduced number of proliferating cells. There are at least two explanations for the latter observation: 1) the release of adult-born neuroblasts from the SVZ into the RMS was delayed; and/or 2) cells generated in the SVZ and RMS after DA depletion survived longer than normal. Arguing against a delayed release of neuroblasts from the SVZ is the normal number of BrdU+ cell bodies in the RMS 6 days after the last BrdU administration. In support of the notion that adult-born cells survive for longer than normal are our findings that despite a reduced number of proliferating cells in the SVZ: 1) most BrdU+ cell bodies in the SVZ were DCX-ir neuroblasts; 2) the rate of neuroblast migration through the RMS was similar to control, with the number of BrdU+ cell bodies throughout the RMS being unaltered 6 days after the last BrdU pulse; and 3) the number of mature BrdU+ interneurons in the GL and GCL that expressed TH- and GABA-LI was greater than control 30 days after the last BrdU pulse.

The three neurochemically identifiable interneuronal populations examined in the GL responded differently to reduced proliferation in the SVZ. 42 days after striatal DA denervation, the number of calbindin-ir cell bodies in the GL was not altered. This suggests that reduced SVZ proliferation in the adult has a limited influence on these cells, most of which are generated early in life [56]. In contrast, calretinin- and TH-ir cell bodies are predominantly generated in the adult [31], [56], [59] and their numbers were affected, with fewer calretinin-ir cell bodies and more TH-ir cells in the GL 42 days after striatal DA denervation. Such plasticity in the population of TH- and calretinin-ir interneurons may reflect their ability to adapt to continuously changing odor environments [60].

A greater number of BrdU+ cell bodies that expressed GABA- and/or TH-LI were observed in the OB of DA-depleted mice 42 days after 6-OHDA administration. Increased numbers of BrdU+/TH-ir cell bodies in the periglomerular region of the OB are consistent with previous observations [19], while to our knowledge, this is the first report of increased BrdU+/GABA-ir cell bodies in the GCL following striatal/SVZ DA depletion. The longevity of adult-born cells therefore appears to have increased, which should contribute to maintaining normal numbers of mature neurons in the GCL and GL of the OB.

Our findings are in contrast to previous reports that used agents that cause permanent and often complete suppression of proliferation in the SVZ, and that examined the effects of reduced SVZ proliferation over a much longer period. Imayoshi and colleagues [61] found a gradual decrease in granule cells from 3–12 weeks following the genetic ablation of newly formed neurons in adult mice. Other recent studies of OB neurogenesis used the antimitotic agent cytosine-*β*-d-arabinofuranoside (Ara-C) and x-ray or gamma-ray irradiation to restrict proliferation in the SVZ [62], [63], [64]. While we found that the number of neuroblasts in the SVZ, RMS and OB was unchanged 7 or 42 days after striatal DA depletion, DCX-ir neuroblasts were almost completely absent following a 28 day infusion of Ara-C [62], or reduced by 70% 7 months following gamma-ray irradiation [65]. Accordingly, the prolonged survival of neuroblasts and GABA and TH cell bodies following partial suppression of proliferation in the SVZ may not be able to maintain a normal number of cells in the OB long-term, and this might become manifest over a longer period of time, by a reduction in migrating neuroblasts in the RMS and mature OB interneurons. This was the case when proliferating cells in adult mice were genetically ablated [61].

The OB contained a normal number of mature OB interneurons, but a greater proportion of mature, adult-born, GABA- and TH-ir interneurons 42 days after striatal DA-depletion. The escalated integration and survival of these adult-born cells, together with changes in subtype of interneuron present in the OB, may alter the complex circuitry that exists within the OB. This includes the intricate arrangement of dendrites in the external plexiform layer that are derived from mitral, granule and tufted cells that engage in dendro-dendritic reciprocal synaptic interactions with each other [66], [67], [68], [69], [70], [71], [72], and the interactions of periglomerular cells in the glomerular layer [73], [74], [75], [76]. Because of the greater proportion of adult-born interneurons making up OB circuitry, functional properties of mitral and tufted cells (e.g. their odorant-evoked firing properties [77]) and the timing of the transmission of olfactory information and bulbar output may be altered. Thus, it would be interesting to determine whether deficits in olfactory functioning, e.g. short- and long-term odor memory, odor discrimination and fear conditioning [43], [62], [65], [78], [79], [80], [81], exist following striatal DA depletion, and whether such deficits are restored in the face of extended survival of adult-born neurons in the OB.

In conclusion, using a model of striatal DA depletion, we show that a moderate reduction of proliferation in the SVZ does not alter the number of mature neurons in the OB, owing to an increased survival of neuroblasts in the SVZ and RMS, and adult-born cells that matured into GABA- or TH-expressing interneurons in the OB. Further investigations are needed to understand the role of these ‘longer-surviving’ adult-born cells on OB circuitry and function.

## Materials and Methods

### Animals

#### Ethics Statement

All experimental procedures performed in this study conformed to the Australian National Health and Medical Research Council published code of practice, and were approved by the Florey Neuroscience Institutes' Animal Ethics Committee (#09-053). 32 male 12-week old C57BL/6 mice weighing between 25–30 g were used, and animals were maintained under standard conditions on a 12 h day/night cycle, with water and food *ad libitum*.

### Lesioning of DA neurons in the substantia nigra pars compacta

Anesthesia was induced in mice by inhalation of 5% isoflurane (Delvet, Seven Hills, NSW, Australia). Mice were then transferred onto a stereotaxic apparatus (Kopf Instruments, Tujunga, CA). Subsequently, anesthesia was maintained with 1.5% isoflurane through a nose cone at the level where a hind paw pinch reflex could not be elicited. 6-OHDA, (Sigma-Aldrich, St. Louis, MO) was used to destroy DAergic neurons in the SNc and their terminals in the striatum and SVZ. 6-OHDA was prepared to a concentration of 2 µg/µl in sterile 0.9% NaCl containing 0.02% ascorbic acid, and was kept on ice until injected. 1.5 µl of the 6-OHDA solution (or 1.5 µl of sterile 0.9% NaCl for control animals) was infused into the right SNc using a glass micropipette attached to a 5 µl Hamilton syringe. Injection coordinates were: 3.0 mm posterior to Bregma, 1.2 mm lateral to the midline and 4.3 mm ventral to the surface of the brain [82], [83]. The injections were delivered over a period of 2 min after which the micropipette was left in position for a further 2 min before being removed at a rate of 1 mm/min. Meloxicam (Troy Laboratories, Smithfield, NSW, Australia, 3 mg/kg i.m.) was administered immediately post surgery for analgesia.

### BrdU administration

BrdU (ICN Biomedicals Inc, Aurora, Ohio, Cat No. 100171) was administered intraperitoneally to 6-OHDA administered and control mice to study the proliferation, migration and survival of adult-born cells in the SVZ, RMS and OB. Three different protocols were used to identify either proliferating cells in the SVZ, migrating cells through the RMS, or mature cells in the OB (Fig. 2): 1) To enable identification of proliferating cells in the SVZ or RMS, a single dose of BrdU (150 mg/kg, i.p.) was injected 2 hours prior to death, 7 or 42 days after 6-OHDA was injected (Fig. 2A); 2) To identify migrating neuroblasts in the RMS and the extent of their migration along the RMS, BrdU (50 mg/kg, i.p.) was administered twice daily for 3 consecutive days beginning 7 days after the 6-OHDA injection, and mice killed 6 days later (i.e. 15 days after 6-OHDA administration) (Fig. 2B); and 3) To label mature adult-born cells that migrate to, integrate and survive in the GCL and GL, BrdU (50 mg/kg, i.p.) was administered twice daily for 5 consecutive days, beginning 8 days after 6-OHDA injections were performed, and mice killed 30 days later (i.e. 42 days after 6-OHDA administration) (Fig. 2C).

### Tissue preparation

All animals were deeply anaesthetized using pentobarbitone sodium (Lethabarb, Virbac, Milperra, NSW, Australia, 100 mg/kg i.p.) and perfused through the heart via the ascending aorta with 20 ml Ca^2+^-free Tyrode's buffer (37°C), followed by 20 ml of a mixture of 4% paraformaldehyde (Sigma) and 0.2% picric acid (Sigma) diluted in 0.16 M phosphate buffer (pH 6.9, 37°C) [84], [85] and 50 ml of the same fixative at 4°C, the latter for approximately 5 min. The brains were dissected out and postfixed in the same fixative for 90 min at 4°C, and finally immersed for 48 h at 4°C in 10% sucrose dissolved in phosphate buffered saline (PBS, pH 7.4) containing 0.01% sodium azide (Sigma) and 0.02% bacitracin (Sigma), before rapid freezing by CO_2_. Sections were cut using a cryostat (Leica CM1850, Wetzlar, Germany) at a thickness of 14 microns, and thaw-mounted on slides coated with 0.5% gelatin- (Sigma) and 0.05% chromium(III) potassium sulphate dodecahydrate (Merck, KGaA, Darmstadt, Germany).

### Immunohistochemistry

#### Incubation protocol (Diaminobenzidine [DAB])

Sections were rinsed (3×10 min) in 0.01 M PBS, followed by incubation in blocking diluent [0.01 M PBS containing 5% normal goat serum (NGS) and 0.3% Triton X-100 (Sigma)] for 30 min, and rabbit anti-Ki67 antibody (1∶15,000, Thermo Fisher Scientific, Fremont, CA, Code No. RM-9106-s1) diluted in 0.01 M PBS, 1% NGS and 0.3% Triton X-100 for 48 hours at 4°C. Sections were then incubated in biotinylated goat anti-rabbit (1∶1,000, Dako, Glostrup, Denmark) diluted in 0.01 M PBS, 1% NGS and 0.3% Triton X-100 for 3 hours at room temperature (RT), and then avidin peroxidase (1∶5000 in 0.01 M PBS and 0.075% Triton X-100) for 1 hour, followed by and nickel-intensified DAB (1∶100, Sigma) for 20 min. 3% Hydrogen peroxidase (Merck) was added to the DAB solution for substrate precipitation and the reaction terminated 2 min later by rinsing sections in 0.01 M PBS. Sections were counter stained with neutral red, dehydrated in a series of graded ethanol (50%–100%), cleared in X3B solvent (Shell Chemicals, Hawthorn East, Australia), and then coverslipped with DePeX (VWR International, Poole, England). Rinses using 0.01 M PBS (3×10 min) were performed between each step.

#### Incubation protocol (Immunofluorescence)

Sections were washed using 0.01 M PBS (3×10 min) and incubated for 24 hours at 4°C with a rat anti-BrdU (1∶300, Axyll, Westbury, NY, Code No. OBT0030), rabbit anti-calbindin (1∶10,000, Swant, Marly, Switzerland, Code No. CB-38a), goat anti-calretinin (1∶4,000, Swant, Code No. CG1), goat anti-doublecortin (1∶1,000, Santa Cruz Biotechnology, Santa Cruz, CA, Code No. SC-8066), rabbit anti-γ-aminobutyric acid (1∶2,000, Sigma, Code No. A2052), rabbit anti-glial fibrillary acidic protein (1∶400, Dako, Code No. Z0334), mouse anti-neuronal nuclei (1∶1,000, Millipore, Billerica, MA, Code No. MAB377), rabbit anti-tyrosine hydroxylase (1∶1,000, Pel-Freeze, Rogers, Ar, Code No. P40101-0) or sheep anti-tyrosine hydroxylase (1∶400, Pel-Freeze, Code No. P60101-0) antibody, diluted in 0.01 M PBS containing 0.3% Triton X-100 and 0.5% BSA. Sections were then washed in TNT buffer [0.1 M Tris-HCl, pH 7.5; 0.15 M NaCl; 0.05% Tween 20 (Sigma)] for 15 min and incubated in TNB buffer [0.1 M Tris-HCl, pH 7.5; 0.15 M NaCl; 0.5% blocking reagent (PerkinElmer, Boston, MA, Code No. FP1020)] for 30 min at room temperature (RT). Immunoreactivity was visualized using Alexa Fluor® 594-conjugated goat anti-rat, Alexa Fluor® 488-conjugated donkey anti-goat, Alexa Fluor® 594-conjugated goat anti-rabbit, Alexa Fluor® 488-conjugated goat anti-rabbit, Alexa Fluor® 594-conjugated goat anti-mouse or Alexa Fluor® 488-conjugated donkey anti-sheep (1∶200, Molecular Probes, Eugene, OR), as appropriate, in TNB buffer for 2 hours. Finally, sections were washed in TNT (3×10 min) and coverslipped using a fluorescent mounting medium (Dako). Hoechst 33342 (1∶1000, Invitrogen, Carlsbad, CA) was applied to sections immunostained with GFAP for 1.5 min during the third TNT wash, to provide a nuclear counter stain. Prior to commencing immunoreactivity for BrdU, antigen retrieval and DNA denaturation was performed, where sections were incubated in 50% formamide (BDH Laboratory Supplies, England) in 0.01 M PBS at 65°C for 2 hours, 2 M HCl for 30 min at 37°C, and 0.1 M sodium borate (Borax®, Sigma, B-3545) buffer for 10 min at RT.

To visualize calbindin and calretinin immunoreactivity, sections were processed using a commercial kit (TSA^+^, NEN Life Science Products, Inc., Boston, MA). Briefly, following 24 hour incubation in primary antisera, sections were washed in TNT buffer (15 min), incubated with TNB buffer (30 min) and incubated with horse-radish peroxidase (HRP)-conjugated swine anti-rabbit (1∶200, Dako, Copenhagen, Denmark) or HRP-conjugated donkey anti-goat (1∶500, Jackson ImmunoResearch Laboratories, West Grove, PA), as appropriate, diluted in TNB buffer for 30 min. Sections were then washed in TNT buffer (3×10 min) and incubated in a biotinyl tyramide-fluoroscein (BT-FITC) conjugate (NEN) diluted 1∶100 in amplification diluent for 10 min at RT, followed by washes in TNT (3×10 min).

### Image Processing

After processing, sections were examined using a Leica DMLB2 fluorescence microscope (Leica, Wetzlar, Germany), equipped with a dark field condenser and epi-polarization, and epifluorescence with appropriate filter combinations, and with objective lenses of ×10 (N.A. 0.45), ×20 (N.A. 0.70), ×40 (N.A. 0.75), ×60 oil (N.A. 1.40), and ×100 oil (N.A. 1.30). Photographs were taken using a Microfire digital camera (2.3A, Optronics, Goleta, CA) attached to the microscope, operated through Picture Frame software (v2.3, Optronics). For confocal analysis, an Olympus FV1000 confocal laser scanning microscope equipped with ×10 (N.A. 0.4), ×20 (N.A. 0.75), ×40 oil (N.A. 1.30) and ×60 oil (N.A. 1.35) objectives was used. The Alexa Fluor® 488 and FITC labeling was excited using the 473 nm diode laser. For the detection of Alexa Fluor® 594, a 559 nm diode laser was used. Z-stack images were captured with multiple images, each separated by a stepwise depth of 0.4 um in the z-plane. Digital images from the microscopy were slightly modified to optimize for image resolution, brightness and contrast using Adobe Photoshop 7.0 software (Adobe Systems Inc., San Jose, CA), so as to best represent the immunohistochemistry observed at the microscope.

### Stereology

For quantification of cell bodies in the SVZ and RMS, the level at which the anterior commissure (AC) converged through the midline (Bregma 0.14 mm [82]) was used as a reference to define the caudal boundary of the SVZ. Serial sections rostral to this point were acquired, with sections between 0–1400 µm rostral to the AC convergence (i.e. Bregma 0.14–1.54 mm, [82]) defined as containing the SVZ, and sections from 1500–4100 µm (i.e. Bregma 1.6–4.2 mm, [82]) regarded as having the RMS. Estimates of the number of proliferating cells, neuroblasts and mature neurons in the SVZ, RMS and OB were made using a fractionator sampling design according to optical dissector rules [86], [87]. Regular predetermined x, y intervals and counting frame dimensions for all estimates were derived by means of a grid program (Stereoinvestigator® v.7.0, MicroBrightField, Williston, VT, viewed through a microscope, Leica) and are outlined in Table 2. In all animals, 14 µm-thick sections, each 560 µm apart (1∶40 series), were analyzed, and guard zones of 3 µm (top) and 1 µm (bottom) were employed. For quantification of proliferating cells and neuroblasts in SVZ, only the lateral wall of the lateral ventricle was analyzed, as these cells are largely absent in the medial and dorsal walls [88]. Coefficients of error and coefficients of variance were used as estimates of precision, with experimental paradigms accepted only when these coefficients were less than 0.1 [89], [90].

**Table 2:** Counting frame dimensions and x, y co-ordinates for estimates of proliferating cells (Ki67, BrdU 2 hours), neuroblasts, migrating cells (DCX, BrdU 5 days), interneurons and mature cells (NeuN, TH, calbindin, calretinin, GABA, BrdU 30 days) in the SVZ, RMS and OB.

Antibody	Region analyzed	Counting frame size (µm)	Fractionator x, y coordinates (µm)
Ki67	SVZ/RMS	30×20	40×100
BrdU (2 hours)	SVZ/RMS	40×40	50×100
BrdU (5 days)	SVZ/RMS	30×30	50×100
BrdU (30 days)	OB	50×50	100×200
BrdU (30 days)	OB	150×150	150×150
DCx	SVZ/RMS	30×20	70×150
NeuN	GCL	20×20	70×300
NeuN	GL	30×30	70×300
TH	GL	40×40	100×400
Calbindin	GL	80×80	100×400
Calretinin	GL	80×80	100×400
Calretinin	GCL	80×80	150×300
GABA	GL	40×40	100×400
GABA	GCL	20×20	100×300
GABA/TH	GL	40×40	100×400
BrdU (30 days)/TH	GL	150×150	150×150
BrdU (30 days)/GABA	GCL	50×50	100×200

### Statistical analysis

Data were analyzed using GraphPad Prism 4 (GraphPad Software, San Diego, CA). All comparisons were conducted by student t-tests and a value of *P*<0.05 was considered statistically significant. Values are expressed as the mean ± SEM.

## Supporting Information

Figure S1 **Dopamine denervation in the striatum and SVZ 42 days following a 6-OHDA-induced lesion of the SNc.** (A) TH-like immunoreactivity (LI) in the intact striatum and SVZ contralateral to the lesioned SNc. (B) Ablation of TH-LI in the SVZ ipsilateral to the lesioned SNc, and near complete denervation in the striatum. LV, lateral ventricle; St, striatum; LS, lateral septal nucleus; TH, tyrosine hydroxylase. Scale bar in A = 200 µm, applies A and B.
(TIF)
