# Supplementary figures and images for: Reduced Proliferation in the Adult Mouse Subventricular Zone Increases Survival of Olfactory Bulb Interneurons
Source: PLoS One. 2012 Feb 21;7(2):e31549. doi: 10.1371/journal.pone.0031549 (PMC3283653; doi:10.1371/journal.pone.0031549)

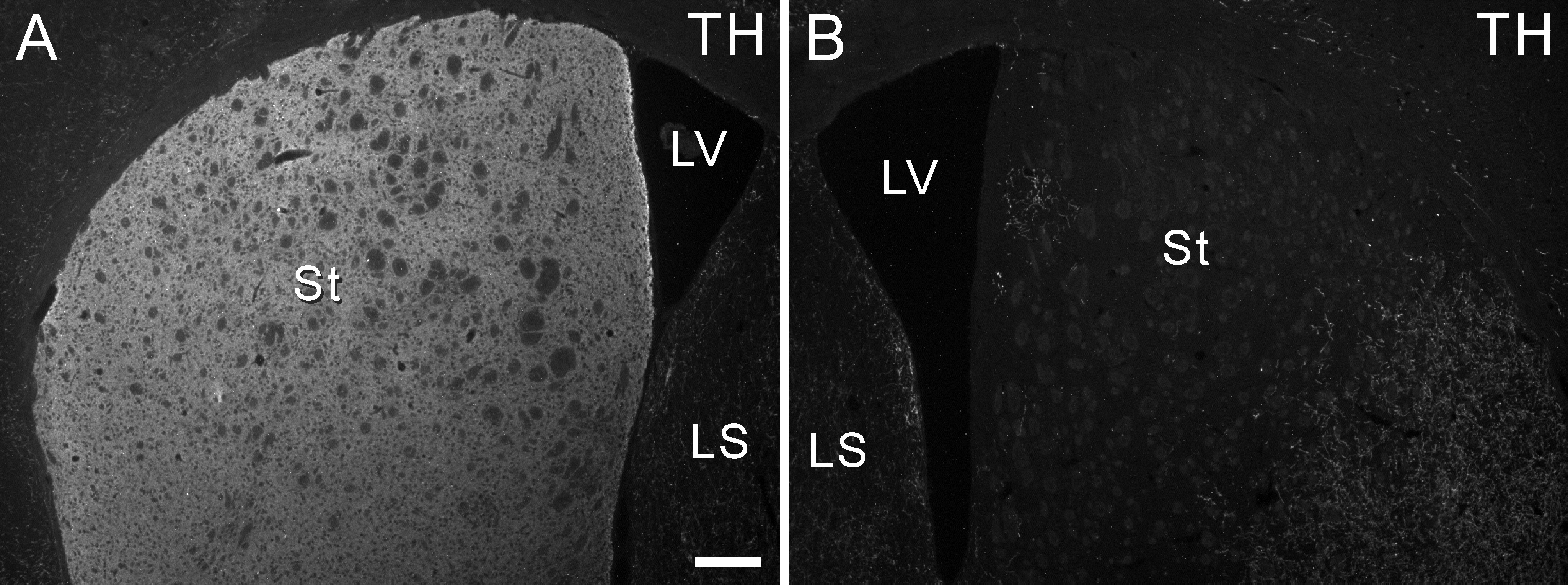

Supplement: Figure S1 — Dopamine denervation in the striatum and SVZ 42 days following a 6-OHDA-induced lesion of the SNc. (A) TH-like immunoreactivity (LI) in the intact striatum and SVZ contralateral to the lesioned SNc. (B) Ablation of TH-LI in the SVZ ipsilateral to the lesioned SNc, and near complete denervation in the striatum. LV, lateral ventricle; St, striatum; LS, lateral septal nucleus; TH, tyrosine hydroxylase. Scale bar in A = 200 µm, applies A and B. (TIF) [file pone.0031549.s001.tif]
